# Association of monoamine oxidase-A genetic variants and amygdala morphology in violent offenders with antisocial personality disorder and high psychopathic traits

**DOI:** 10.1038/s41598-017-08351-w

**Published:** 2017-08-29

**Authors:** Nathan J. Kolla, Raihaan Patel, Jeffrey H. Meyer, M. Mallar Chakravarty

**Affiliations:** 1 Centre for Addiction and Mental Health (CAMH), Psychiatry, Toronto, M5T 1R8 Canada; 2 CAMH, Research Imaging Centre, Toronto, M5T 1R8 Canada; 3 CAMH, Violence Prevention Neurobiological Research Unit, Toronto, M5T 1R8 Canada; 4 Douglas Mental Health University Institute, Cerebral Imaging Centre, Montreal, H4H 1R3 Canada; 5 McGill University, Department of Biological and Biomedical Engineering, Montreal, H3A 2B4 Canada; 6 McGill University, Department of Psychiatry, Montreal, H3A 1A1 Canada

## Abstract

Violent offending is elevated among individuals with antisocial personality disorder (ASPD) and high psychopathic traits (PP). Morphological abnormalities of the amygdala and orbitofrontal cortex (OFC) are present in violent offenders, which may relate to the violence enacted by ASPD + PP. Among healthy males, monoamine oxidase-A (MAO-A) genetic variants linked to low *in vitro* transcription (MAOA-L) are associated with structural abnormalities of the amygdala and OFC. However, it is currently unknown whether amygdala and OFC morphology in ASPD relate to MAO-A genetic polymorphisms. We studied 18 ASPD males with a history of violent offending and 20 healthy male controls. Genomic DNA was extracted from peripheral leukocytes to determine MAO-A genetic polymorphisms. Subjects underwent a T1-weighted MRI anatomical brain scan that provided vertex-wise measures of amygdala shape and surface area and OFC cortical thickness. We found that ASPD + PP subjects with MAOA-L exhibited decreased surface area in the right basolateral amygdala nucleus and increased surface area in the right anterior cortical amygdaloid nucleus versus healthy MAOA-L carriers. This study is the first to describe genotype-related morphological differences of the amygdala in a population marked by high aggression. Deficits in emotional regulation that contribute to the violence of ASPD + PP may relate to morphological changes of the amygdala under genetic control.

## Introduction

Antisocial personality disorder (ASPD), especially when high psychopathic traits are also present (ASPD + PP), is associated with increased risk of violence^1^. Psychopathic traits include features of narcissism, manipulation, and self-aggrandizement^2^. Specifically, individuals with ASPD engage in high rates of assaultive behavior toward their intimate partners and strangers^3, 4^. Given that the prevalence of ASPD approximates 7% in the community^5^ and 50% in prison settings^6^, the investigation of biomarkers underlying the aggression in ASPD + PP is required to better understand the neurobiological underpinnings of this common and clinically important psychiatric condition. An important line of investigation that has yet to be pursued is examination of the relationship between gene function and morphology of brain regions relevant to expression of violence in ASPD + PP.

One such brain region, the amygdala, has become an attractive neuroimaging target in the study of ASPD + PP, since many of its functions, including its involvement in moral reasoning, stimulus-reinforcement learning, and harm avoidance^7–9^, show impairment in persons endorsing high psychopathic traits^10^. At least two structural magnetic resonance imaging (MRI) studies using surface-based mesh modelling techniques have reported abnormal amygdala morphology in samples with high levels of psychopathy^11, 12^. One investigation described tissue enlargement of the lateral and central nuclei in psychopathy, which was accompanied by reduction effects in the basolateral nucleus^11^. The other study reported bilateral amygdala volume loss and atrophy of cortical, central, basolateral, and lateral nuclei that showed a relation with increased psychopathic traits in a predominantly male sample with high psychopathy features^12^. These studies highlight abnormal amygdala morphology as a potential biomarker of elevated psychopathic traits, but they do not provide information on possible genetic factors influencing this brain phenotype.

Another brain region that has been implicated in the pathogenesis of ASPD + PP is the orbitofrontal cortex (OFC)^13^. The OFC contributes to emotion processing, moulds personality structure, and regulates social conduct and behavior^14^. One study found increased bilateral OFC grey matter volumes in male carriers of MAOA-L versus MAOA-H^15^, whereas another investigation reported that the MAOA-L genotype was associated with grey matter loss in bilateral OFC^16^. Importantly, both of these studies were conducted in healthy individuals. In addition, OFC volume loss in a sample of individuals with cocaine use disorders was attributed to the low-activity MAO-A allele in combination with lifetime cocaine use^17^. Tissue reductions have also been reported in the OFC of psychopathy^11^. The OFC is a logical structure to study in tandem with the amygdala, as reciprocal connections exist between the two regions in primates^18^. Moreover, altered white matter connections between the amygdala and OFC have also been described in psychopathy^19^.

Monoamine oxidase-A (MAO-A) is an enzyme that degrades neurotransmitters, such as serotonin, norepinephrine, and dopamine, that are known to play a role in aggressive behavior^20^. Important to the current study is that MAO-A may influence amygdala and OFC morphology. The MAO-A gene includes a 30-base pair variable nucleotide tandem repeat (VNTR) polymorphism that yields variants associated with low (MAOA-L) or high (MAOA-H) transcription efficiency^21^. In some studies, individuals with the MAOA-L genotype exhibit elevated antisocial traits^22^ and, in offender samples, show evidence of severe violent behavior. The MAOA-L allele may also influence amygdala reactivity to the expression of anger^23, 24^. Interestingly, a positron emission tomography investigation of ASPD found that MAO-A activity was lower in the OFC and regions that make direct connections with the amygdala, such as the hippocampus and thalamus^25^.

Previous findings linking MAO-A with alterations in amygdala/OFC volume and morphology have yielded mixed results. At least one study found that MAOA-L carriers display reduced amygdala volumes bilaterally relative to MAOA-H carriers^15^. By contrast, another investigation reported no difference in amygdala volume by MAO-A genotype^26^. The wide age range (18–80 years) of the second sample compared with the younger age of the participants tested in the first study was suggested as one reason for the discrepant results. One study found increased bilateral OFC grey matter volumes in male carriers of MAOA-L versus MAOA-H^15^, whereas another investigation reported that the MAOA-L genotype was associated with grey matter loss in bilateral OFC^16^. Importantly, both of these studies were conducted in healthy individuals. In addition, OFC volume loss in a sample of individuals with cocaine use disorders was attributed to the low-activity MAO-A allele in combination with lifetime cocaine use^17^. Tissue reductions have also been reported in the OFC of psychopathy. However, to the best of our knowledge, no study has ever investigated amygdala or OFC morphology in relation to the MAO-A VNTR polymorphism among a clinical group (ASPD) presenting severe symptoms (PP).

Since MAOA-L shows a relationship with violent behavior and has also been linked to amygdala/OFC structure, the principal aim of the study was to investigate whether the low-activity MAO-A variant was associated with amygdala/OFC volume and morphology in ASPD + PP with a history of violence. We hypothesized that a gene-diagnosis interaction would emerge, such that ASPD + PP with MAOA-L would exhibit brain changes not seen in healthy subjects with the same genotype. We did not expect to find group differences amongst the MAOA-H carriers.

## Results

### Subject Characteristics

Participants ranged in age from 18 to 50 years. ASPD + PP subjects had higher rates of past substance use disorders (Table 1). As expected, ASPD + PP participants endorsed greater PCL-R total and facet scores and presented significantly more conduct disorder and ASPD symptoms.

**Table 1:** Clinical and Demographic Characteristics.

	ASPD MAOA-H	ASPD MAOA-L	Control MAOA-H	Control MAOA-L	Statistics	p-value
Sample Size (*n*)^a^	9	9	11	9	χ^2^ = 0.095	0.76
Age^b^	36.2 ± 8.7	35.7 ± 10.1	37.1 ± 7.8	31.7 ± 6.6	χ^2^ = 4.1	0.25
Ethnicity^c^						
Caucasian	3	4	3	5	/	0.68
Black	2	3	2	0	/	
Asian	1	1	4	3	/	
Hispanic	1	0	0	0	/	
Middle Eastern	1	0	0	1	/	
Aboriginal	1	1	1	0	/	
Other	0	0	1	0	/	
IQ^b^	107.9 ± 13.0	103.6 ± 9.4	112.5 ± 8.1	112.5 ± 8.4	χ^2^ = 4.8	0.19
History of SUD^c^	3	4	0	0	/	0.0049
Total PCL-R score^b^	25.4 ± 7.2	25.6 ± 6.0	3.2 ± 3.1	3.0 ± 2.5	χ^2^ = 26.4	0.000
PCL-R Facet 1 interpersonal score^b^	3.7 ± 1.5	3.7 ± 1.9	0.8 ± 1.0	0.1 ± 0.3	χ^2^ = 25.1	0.000
PCL-R Facet 2 affective score^b^	6.0 ± 1.9	4.8 ± 1.9	0.4 ± 0.7	0.3 ± 0.5	χ^2^ = 27.7	0.000
PCL-R Facet 3 lifestyle score^b^	6.9 ± 2.1	7.9 ± 1.8	1.2 ± 2.0	1.4 ± 1.9	χ^2^ = 25.7	0.000
PCL-R Facet 4 antisocial score^b^	6.4 ± 2.5	7.4 ± 2.5	0.2 ± 0.4	0.2 ± 0.4	χ^2^ = 28.2	0.000
Number of ASPD symptoms^a^	5.5 ± 1.5	5.4 ± 1.0	0	0	χ^2^ = 29.9	0.000
Number of CD symptoms^a^	6.8 ± 4.2	7.7 ± 3.3	0	0.1 ± 0.3	χ^2^ = 29.6	0.000

ASPD = antisocial personality disorder; CD = conduct disorder; IQ = intelligence quotient; MAOA-H = high activity monoamine oxidase-A allele; MAOA-L = low activity monoamine oxidase-A allele; PCL-R = Psychopathy Checklist-Revised; SUD = substance use disorder; a = chi-square test; b = Kruskal-Wallis test; c = Fisher’s exact test.

Table 1 outlines the clinical and demographic information for the four study groups.

### Amygdala Volume

**Figure 1:**
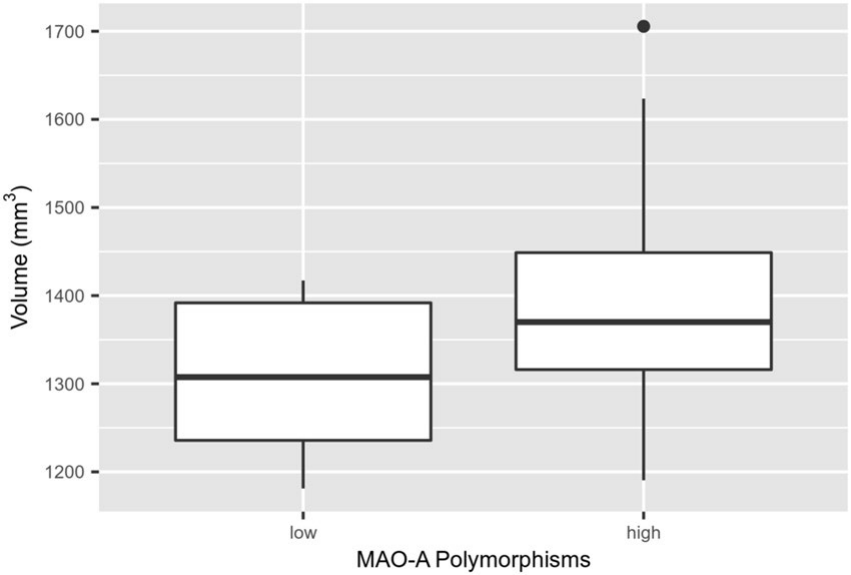
Plot displays the main effect of monoamine oxidase-A (MAO-A) genotype on right amygdala volume, (F(1, 31) = 5.4, *p* = 0.027). Subjects with the low activity MAO-A (MAOA-L) genotype showed decreased right amygdala volume in comparison to subjects with the high activity MAO-A (MAOA-H) genotype (Tukey’s Honestly Significant Difference [HSD] post hoc result = 0.027).

The two-way analysis of variance (ANOVA) interaction was not significant for either the left or right amygdala volume (left: F(1, 31) = 0.3, *p* = 0.58; right: F(1, 31) = 0.7, *p* = 0.39). Similarly, there was no main effect of group for either side (left: F(1, 31) = 0.8, *p* = 0.39; right: F(1, 31) = 0.2, *p* = 0.62). There was a main effect of MAO-A genotype on right amygdala volume (F(1, 31) = 5.4, *p* = 0.027). Specifically, subsequent pairwise Tukey’s Honestly Significant Difference (HSD) post hoc tests revealed that subjects with MAOA-L had decreased volumes (*p* = 0.027) (Fig. 1). There was no group effect on the left amygdala (F(1, 31) = 2.1, *p* = 0.16).

### Amygdala Surface Area

**Figure 2:**
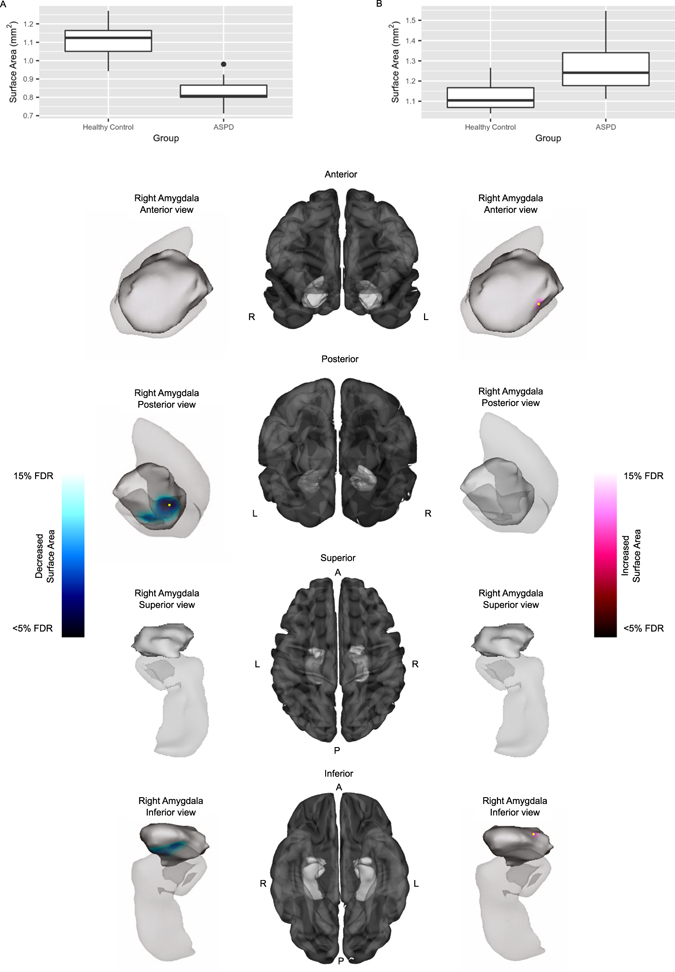
Surface area differences in antisocial personality disorder with high psychopathic traits (ASPD + PP) and controls with MAOA-L genotype. Anterior, posterior, superior, and inferior views of the right amygdala and hippocampus (hippocampal surface provided for context only) are shown in the left and right panels, with significant surface area decreases in the right basolateral (BLA) nucleus (left panel) and significant increases in the right anterior cortical amygdaloid (ACo) nucleus on the right panel. Cortical representations in the center panel are provided for further context. Plots (**A**) and (**B**) display the surface area measurements at a peak vertex denoted by the yellow markers to demonstrate the nature of the effect. ASPD + PP offenders showed decreased surface area in the right BLA nucleus of the amygdala (posterior, inferior views, plot (**A**) as well as increased surface area in the right anterior ACo nucleus, (F(1, 13 = 6.82, 15% false discovery rate [FDR]) (anterior, inferior views, plot (**B**)).

A group × genotype interaction was present in the right amygdala (F(1, 31) = 11.47, corrected at 15% false discovery rate [FDR]). Specifically, ASPD + PP offenders with the MAOA-L genotype showed decreased surface area in the right basolateral (BLA) nucleus and increased surface area in the right anterior cortical amygdaloid (ACo) nucleus versus MAOA-L genotype controls (F(1, 13) = 6.82, corrected at 15% FDR) (Fig. 2). BLA and ACo nuclei were identified as the locations of morphological change using visual comparison to a reference atlas (see Methods). Results did not survive a 15% FDR threshold when comparing ASPD + PP subjects with MAOA-H to MAOA-H healthy subjects.

### Psychopathy Checklist-Revised Scores and Amygdala Surface Area

**Figure 3:**
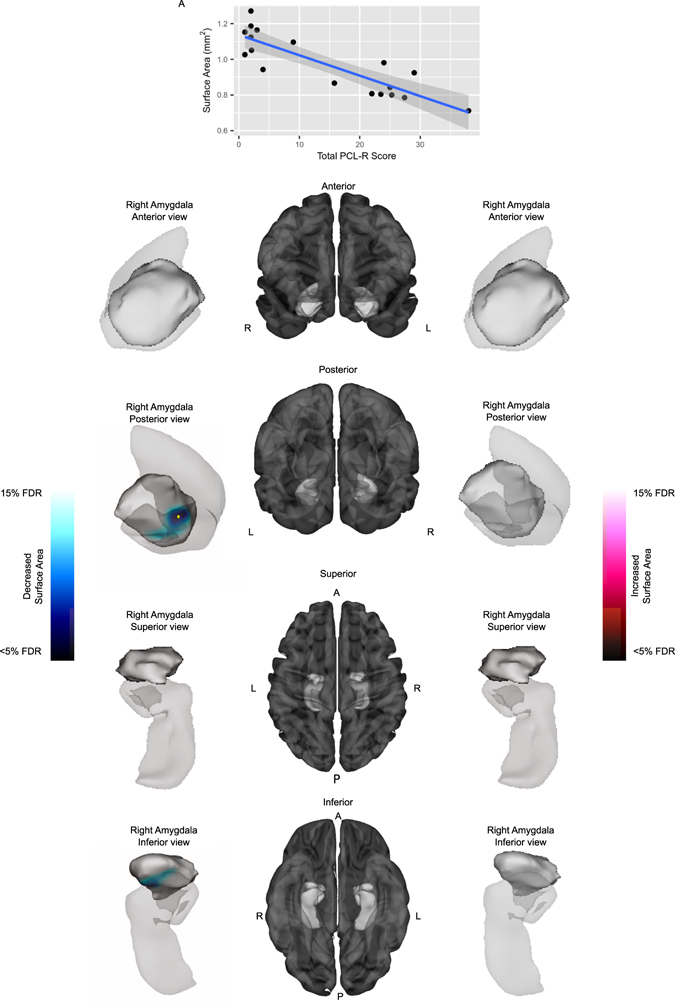
Relationship between PCL-R scores and amygdala surface area among individuals with the MAOA-L genotype. Anterior, posterior, superior, and inferior views of the amygdala and hippocampus (provided for context) are shown in the left and right panels, with a significant negative correlation shown on the left. Cortical representations in the center panel are provided for further context. Plot (**A**) displays the surface area measurements at a peak vertex denoted by the yellow markers. PCL-R score showed a negative correlation with surface area in the right BLA nucleus of the amygdala, (F(1, 13) = 6.55, 15% FDR) (posterior, inferior views, Plot (**A**)).

In an exploratory analysis among subjects with MAOA-L, Psychopathy Checklist-Revised (PCL-R) scores were found to have a significant effect on right amygdala surface area (F(1, 13) = 6.55, corrected at 15% FDR). Specifically, PCL-R scores showed an inverse correlation with surface area in the right BLA nucleus (Fig. 3).

### OFC Cortical Thickness

**Figure 4:**
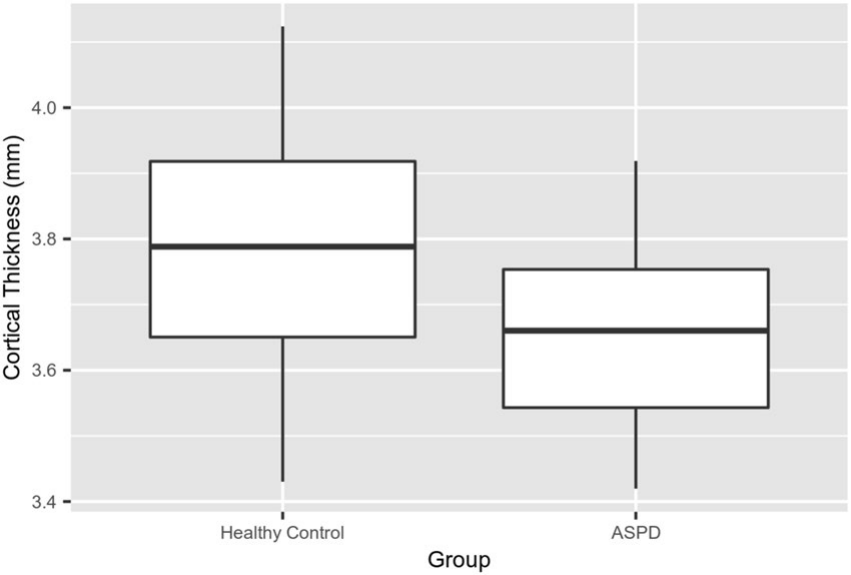
Plot displays a main effect of group in cortical thickness in the left lateral orbitofrontal gyrus, (F(1, 31) = 5.13, *p* = 0.031). Cortical thickness for the region of interest (ROI) was computed on a per subject basis via application of the LPBA40 atlas to the cortical surface of each subject. Thickness measures across the ROI were then averaged to output a mean cortical thickness per ROI for each subject. In this comparison, ASPD + PP offenders showed decreased cortical thickness in the left lateral orbitofrontal gyrus (Tukey’s HSD post hoc test = 0.031).

The two-way interaction was not significant for the middle orbitofrontal gyrus (left: F(1, 31) = 0.02, *p* = 0.88; right: F(1, 31) = 0.14, *p* = 0.72) or lateral orbitofrontal gyrus (left: F(1, 31) = 0.13, *p* = 0.73; right: F(1, 31) = 0.98, *p* = 0.33). A main effect of group was present in the left lateral orbitofrontal gyrus (F(1, 31) = 5.13, *p* = 0.031) but not the left middle orbitofrontal gyrus (F(1, 31) = 4.0, *p* = 0.054). Tukey’s HSD post hoc tests indicated that the ASPD + PP group had decreased cortical thickness in the left lateral orbitofrontal gyrus compared with healthy controls (*p* = 0.031) (Fig. 4). There was no group effect for cortical thickness in the right middle orbitofrontal gyrus (F(1, 31) = 2.2, *p* = 0.14) or the right lateral orbitofrontal gyrus (F(1, 31) = 1.2, *p* = 0.28). Similarly, there was no main effect of MAO-A genotype for cortical thickness in the middle orbitofrontal gyrus (left: F(1, 31) = 0.2, *p* = 0.64; right: F(1, 31) = 1.8, *p* = 0.19) or lateral orbitofrontal gyrus (left: F(1, 31) = 0.1, *p* = 0.79; right: F(1, 31) = 0.6, *p* = 0.43).

### Amygdala Surface Area Power Analysis

**Figure 5:**
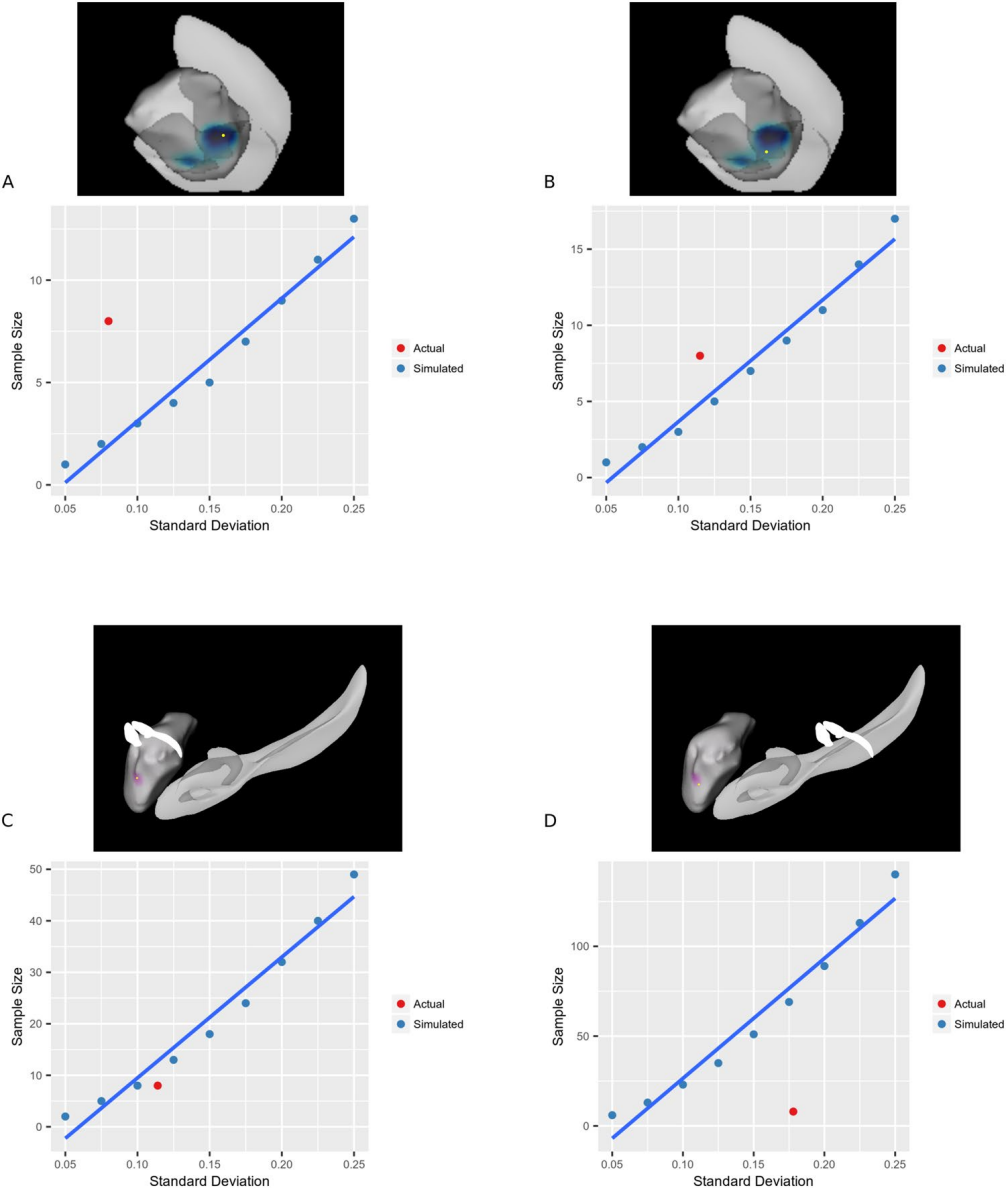
Plots of standard deviation versus required sample size for power = 0.8 and α = 0.05. At each vertex, the sample size was calculated that would be required to obtain 80% power given α = 0.05 and a variety of standard deviations. These sample sizes are plotted in blue as simulated data. In red, the actual standard deviation was plotted at each vertex versus the sample size of each group (8), allowing a comparison of our sample size versus the sample size required to measure the effect. This procedure was performed for four separate vertices. Plots (**A**) and (**B**) show data from vertices where ASPD subjects showed significantly decreased surface area; plots (**C**) and (**D**) show vertices where significantly increased surface area was observed in ASPD. Plots (**A**) and (**C**) correspond to “strong vertices,” where the effect passed 15% FDR easily, while (**B**) and (**D**) correspond to “weak vertices,” where the effect barely passed 15% FDR. The goal of this experiment was to examine the full breadth of observed effects. In (**A**), (**B**), and (**C**), the actual sample size of 8 is at least sufficient for observing the effect given the standard deviation of surface area measurements at the corresponding vertex. In (**D**), the actual sample size of 8 would not have been sufficient to achieve 80% power given the observed standard deviation. Overall, plots (**A**–**D**) give confidence that the sample size of the study is sufficient to observe the reported effects.

Results above indicated that ASPD + PP offenders with MAOA-L had different right amygdala surface areas in two regions (BLA and ACo) than healthy controls with MAOA-L. Two vertices were selected for each region. At each vertex, the mean surface area of the two groups and a range of variances were used to simulate the required sample size for a study with power = 0.8 and α = 0.05. These simulated required sample sizes were then plotted against the actual sample size and variance of surface area at the given vertex. In three of four vertices, the sample size of our study was sufficient to meet the requirements, suggesting that the sample was large enough to reliably detect the observed effects (Fig. 5).

### OFC Cortical Thickness and Power Analysis

**Figure 6:**
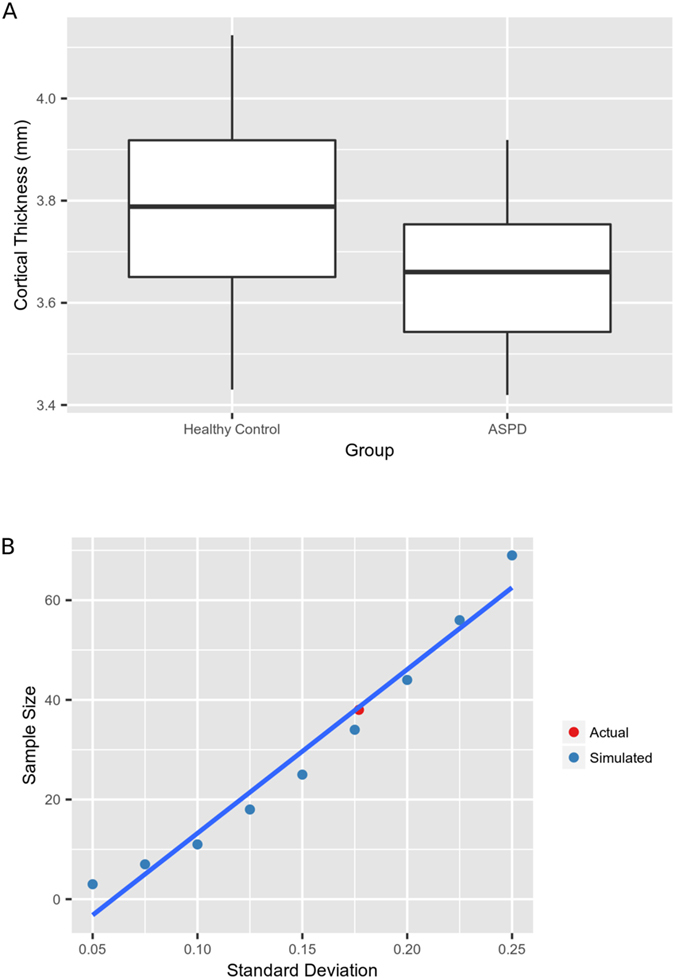
Plots of standard deviation versus required sample size for power = 0.8 and α = 0.05. For the left lateral orbitofrontal gyrus, we calculated the sample size that would be required to obtain 80% power given α = 0.05 and a variety of standard deviations. These sample sizes are plotted in blue as simulated data. In red, the actual standard deviation of cortical thickness in each ROI versus the sample size of each group was plotted (8), allowing a comparison of our sample size versus the sample size required to measure the effect. Plot (**A**) depicts data from the left lateral orbitofrontal gyrus and plot (**B**) shows the corresponding plot of standard deviation versus required sample size. The actual sample size falls within a reasonable range of that required to observe the effect in a study with power = 0.8 and α = 0.05, giving confidence that the sample size of our study is sufficient to observe the reported effects.

Results above indicated that ASPD + PP offenders had less cortical thickness in the left lateral orbitofrontal gyrus. The mean cortical thickness of the two groups and a range of variances were used to simulate the required sample size for a study with power = 0.8 and α = 0.05. These simulated required sample sizes are plotted against the actual sample size and variance of the cortical thickness measure. The plot shows that the sample size was sufficiently large enough to reliably detect the observed effects (Fig. 6).

## Discussion

This investigation is the first to demonstrate that MAO-A VNTR genetic polymorphisms show a relation with amygdala morphology in ASPD + PP. Our most noteworthy finding is that ASPD + PP offenders with the MAOA-L genotype exhibited decreased surface area of the right BLA nucleus and increased surface area of the right ACo nucleus. Another finding is that these alterations were associated with increased psychopathic traits. Previous studies of ASPD + PP reported amygdala surface area anomalies but did not compare results with additional biological measures^11, 12^. By contrast, our investigation included genetic analyses, whose results suggest that the observed morphological abnormalities in ASPD + PP may relate to MAOA-L genotype effects. We believe that the results of the present investigation are significant, because they suggest a novel mechanism to help explain the morphological changes present in the amygdala of ASPD + PP.

One of our two main results was a reduction in BLA nucleus surface area among MAOA-L carriers with ASPD + PP versus healthy subjects matched on genotype. The finding of decreased BLA nucleus surface area in ASPD + PP agrees with previous work that reported reduction effects in this region^11^, and it adds to a growing body of evidence linking abnormalities of the BLA nucleus to key cognitive impairments in psychopathy. Individuals with high trait psychopathy are insensitive to punishment and exhibit weakened autonomic responding to fearful or threatening stimuli^27^. Interestingly, acquisition of fear conditioning depends on intact microcircuitry within the BLA, such that the fear-conditioned startle response to conditioned stimuli is abolished in rodents with lesioned BLA^28, 29^. Because serotonin is an important neuromodulator of BLA neurons^30^, genetic influences that regulate serotonin neurotransmission may affect the integrity of the BLA nucleus. We suggest that alteration of the BLA nucleus in ASPD + PP could relate to the effects of the MAOA-L genetic variant and its regulation of serotonergic neurotransmission in the developing amygdala. This position is strengthened by the observation of anomalous accumulation of serotonin in the amygdala of MAO-A knockout models^31^. Our model linking emotional deficits in psychopathy to altered monoamine functioning in the amygdala accords well with studies indicating preferential binding of MAO-A inhibitors to rodent BLA^32^ and reduced fear conditioning in healthy subjects administered selective serotonin reuptake inhibitors^33^.

A second main finding is that the right ACo nucleus surface area was increased in ASPD + PP offenders carrying the MAOA-L genotype. A primary function of the ACo is to process smell directly from the main olfactory bulb^34^. Interestingly, the higher olfactory processes of identification and discrimination operate less efficiently among individuals with elevated psychopathic traits^35^. The notion that impaired sense of smell can be attributed to focal brain changes is congruent with clinical observations of faulty olfaction in other neurological disorders such as Parkinson’s disease^36^. These neural alterations may also relate to the effect of specific MAO-A genotypes impacting sensory perception in vulnerable individuals. One consequence of reduced MAO-A activity that stems from the MAOA-L genotype may be increased dopaminergic neurotransmission. In the mouse main olfactory bulb, surges in dopamine impair detection of social odors localized to male urine^37^. Dopamine levels were additionally shown to have increased in the nucleus accumbens after rats engaged in physically aggressive confrontations with conspecifics^38^. Therefore, lowered MAO-A may link olfactory impairments in high psychopathy with physical aggression, perhaps through increased limbic dopamine transmission. Indeed, lower prefrontal MAO-A binding predicted greater anger/hostility in a PET study of healthy human subjects^39^.

The fact that differences in the surface area compartments of the amygdala were not paralleled by changes in amygdala volume suggests that the observed morphological differences may have been due to alterations in dendritic arborization as opposed to alterations in actual number of neurons. Pre-clinical research has revealed that MAO-A knockout (KO) mice and a hypomorphic mutant mice strain characterized by reduced transcription of functional MAO-A exhibit decreased dendritic arborization of the basolateral amygdala relative to wild-type^40, 41^. On the other hand, in a serotonin transporter (5-HTT) KO model, apical dendritic length and spine density in the BLA nucleus were increased in the mutant strain relative to wildtype^42^. Similarly, wild type mice subjected to a chronic stress paradigm produced hypotrophy of dendritic aborization and greater spine density in the BLA nucleus^43^. While the first two studies speak to possible genetic influences impacting dendritic arborization and the development of ASPD phenotype in males, the third study describes environmental exposures that may guide the assembly of dendritic morphology important to expression of ASPD. This last point is relevant, since individuals with ASPD are known to manifest low distress tolerance^44^. We were unable to locate any studies describing an association between MAO-A VNTR genotype and amygdala dendritic arborization, nor were we able to find research that genetic or environmental influences affect arborization in the ACo nucleus.

It is likely that morphological changes of the amygdala in ASPD are related to other factors that may interact with genetic susceptibility. The serotonin system may represent one potential avenue. MAO-A KO mice exhibit increased serotonin levels in the amygdala and also display overt aggressive behavior^31^. Furthermore, pharmacological blockade of amygdala 5-HTT receptors has been shown to reduce aggression in MAO-A knockout models^45^. Other environmental factors may also shape the outcome of high serotonin levels that confer vulnerability to antisocial traits. For example, early childhood adversity is associated with altered serotonin systems in humans^46^ that may be due to epigenetic modification of serotonin genes^47^ and/or the reciprocal interaction that exists between the serotonergic system and the hypothalamopituitary axis^48, 49^. Although ample evidence suggests that MAOA-L and early childhood adversity may interact to produce antisocial outcomes^50^, the literature is silent on whether the combination of these variables may alter amygdala morphology.

We did not detect any significant gene-diagnosis interactions in the OFC. We did ascertain, however, that ASPD + PP participants displayed smaller OFC volumes than the healthy controls, which accords well with findings in the literature^51, 52^. Interestingly, a small voxel-based morphometry study comprising a non-clinical cohort of males with relatively high psychopathic traits did not report an interaction between MAOA-L and group in the OFC, although they did find grey matter reductions in the right superior temporal pole^53^. Given these findings, it may be that the OFC in early development is less sensitive to the effects of MAO-A VNTR genetic polymorphisms than the amygdala.

We note several limitations of the present study. First, the sample size was relatively small. However, as this study is the only neuroimaging investigation in an antisocial population that has, to the best of our knowledge, considered the effect of genotype on structural brain changes, we are emboldened by these novel results. We have further confidence in our findings, as our power analysis indicated that we were adequately powered to detect significant results given the effect sizes observed. Second, the imaging findings could reflect the influence of genetic variants other than MAO-A that are in linkage disequilibrium with MAO-A. However, the fact that we obtained signal from regions showing abundant MAO-A expression increases our confidence that we are observing activation of the MAOA system. Third, the observed effects could also relate to a range of other non-genetic influences. On the other hand, we measured and controlled for a host of clinical variables that could have impacted our results. Fourth, it is also possible that it was the effect of the MAOA-L genotype which exposed the individual to the influences that made him engage in antisocial behavior. However, not all MAOA-L males had ASPD and some of the typically developing group were also of this genotype. Moreover, the experimental group included some MAOA-H carriers with ASPD as well as typically developing individuals with MAOA-H or MAOA-L.

In summary, we demonstrated that ASPD + PP with MAOA-L display decreased surface area in the ri﻿ght BLA and increased surface area in the right ACo. We take the position that these alterations in the amygdala were influenced by the effect of MAOA-L. Future investigations examining the neurobiological substrate of ASPD + PP should pay heed to the genetic make-up of this group to better determine whether other genotypes implicated in violent behavior also impact brain structure and function in ASPD.

## Methods

### Participants

Thirty-eight males completed the study protocol: 18 participants with ASPD + PP and 20 healthy controls. Subjects provided written consent to take part in the investigation after study procedures were explained to them. All components of the study were approved by the Research Ethics Board for Human Subjects at the Centre for Addiction and Mental Health, Toronto, Ontario. All methods were performed in accordance with the relevant guidelines and regulations.

### ASPD Participants

Subjects with ASPD + PP were recruited from the community and from half-way houses operated by correctional services. One forensic psychiatrist (NJK) clinically assessed and diagnosed participants with ASPD using the Structured Clinical Interview for DSM IV Axis II Disorders (SCID-II)^54^ and also used the Structured Clinical Interview for DSM IV-TR Axis I Disorders (SCID-I)^55^ to assess for other psychiatric illness. Each participant had a history of violent offending that included charges of assault, sexual assault, robbery, uttering threats, and manslaughter. A history of major depressive disorder, bipolar disorder, or a schizophrenia spectrum disorder was exclusionary. Current use of illicit substances, cigarette smoking^56^, and use of psychotropic medications were also exclusionary. To verify the non-smoking status of subjects, breathalyzer testing for carbon monoxide was conducted (MicroSmokerlyzer; Bedfont Scientific Ltd., Kent, United Kingdom). Subjects additionally provided negative urine toxicology screens on all scanning and assessment days.

### Control Participants

Healthy participants responded to recruitment ads seeking study participants. These ads were placed in the hospital, community, and on websites. Control subjects were also recruited and administered the SCID-I and SCID-II by NJK. Control subjects had no lifetime history of psychiatric disorder. All were non-smokers, as determined by self-report and breathalyzer testing, and urine toxicology screens were uniformly negative throughout the study. Criminal record checks from federal government agencies determined that none of the control participants had criminal charges.

### Additional Inclusion and Exclusion Criteria

All ASPD and control participants were right-handed. None had a history of neurological illness, and those with a history of head trauma resulting in loss of consciousness were excluded from participation.

### PCL-R

All subjects were administered the Psychopathy Checklist – Revised (PCL-R)^57^ by a trained forensic psychiatrist (NJK). The PCL-R is a clinical instrument that operationalizes psychopathy based on 20 personality and behavioral items. Each item is rated from 0 to 2 based on whether the trait is present (0 = absence of trait; 1 = some indicators of the trait; 2 = definite presence of the trait). Total PCL-R scores range from 0 to 40. Four facet scores can also be generated from the PCL-R that reflect different aspects of prototypical psychopathy. Facet 1 describes interpersonal features; facet 2 indexes affective components of psychopathy; facet 3 assesses lifestyle items; and facet 4 relates to antisocial behavior.

### ASPD and Conduct Disorder Symptoms

The number of ASPD and conduct disorder symptoms were obtained from the SCID-II interview.

### Past Substance Misuse

Diagnoses of substance use disorders in remission were obtained from the SCID-I interview.

### Intelligence

The Wechsler Test of Adult Reading^58^ was administered to all participants to provide an estimate of full-scale IQ.

### Genetics

Standard PCR procedures using primers as previously described were used to amplify the MAO-A VNTR locus^59^. We implemented minor changes, including labeling the forward primer with 5′ HEX modifier that allowed for electrophoresis and visualization on a capillary sequencer. In brief, 125 ng of total genomic DNA was combined with the following elements: 1X PCR_x_ Amplification Buffer, 1.5 mM MgSO_4_, and 1X PCR_x_ Enhancer Solution that accompanied the Invitrogen^TM^ PCR_x_ Enhancer Kit, 0.2 mM of each dNTP, 0.0975 ug of each primer, and 0.5 U Taq polymerase. A total reaction volume of 20 uL was produced. The cycling conditions were the same as previously described^59^, save for a further denaturation step of 5 min at 95 °C. The ABI 3130 Genetic Analyzer system and GeneMapper software (ThermoFisher Scientific, Waltham, MA) were used to electrophorese and visualize 1 µL of the amplified product. Subjects with 2, 3, or 5 copies of the MAO-A VNTR were assigned the MAOA-L genotype, while individuals with 3.5 or 4 copies were designated as MAOA-H carriers.

### Image Acquisition

Each subject received a T1-weighted anatomical scan for the region of interest analysis (TR = 6.7 ms, TE = 3.0 ms, flip angle = 8°, slice thickness = 0.9 mm, 200 slices, matrix = 256 × 256, FOV = 250 mm, voxel size = 0.9 mm × 0.9 mm × 0.9 mm) performed on a 3.0-T GE Discovery MR750 scanner (GE Medical Systems, Milwaukee, WI).

### MRI Image Processing

All T1-weighted MRI data were converted to the MINC file format (http://www.bic.mni.mcgill.ca/ServicesSoftware/MINC) and underwent preprocessing using the bpipe tools from the CoBrA Laboratory (https://github.com/CobraLab/minc-bpipe-library). This process included rigid (6-parameter) registration to MNI space^60^, cropping of the neck region to improve downstream image processing, N4 correction of bias field intensity inhomogeneity^61^, and brain extraction using BeAST^62^.

### Amygdala Volume and Morphometry

These parameters were assessed using the MAGeT Brain pipeline (http://cobralab.ca/software/MAGeTbrain/)^63^ based on previous neuroanatomical definitions of the amygdala derived by our group^64^. In brief, five high-resolution atlases with amygdala segmentation were used as inputs^65^. MAGeT Brain bootstraps the segmentation by using an intermediate template library of 21 subjects sampled from the entire population under study and chosen to represent that variability with respect to age and sample distributions (e.g., case vs. control status). Each template receives five segmentations via model-based segmentation with each of the atlases. Finally, at the subject-level, each subject undergoes model-based segmentation with each of the templates, thereby growing the number of possible candidate labels to 105. These labels are then fused via majority-vote to generate the final segmentation. Surface-based representations of the amygdala were generated using a similar methodology as previously described^66, 67^, where coordinates for developing the final surface were generated based on the median location of the 105 possible surfaces. Vertex-wise measures of surface area were generated by averaging the individual areas of the three polygons at the intersection of each vertex and were blurred using a 5 mm surface-based blurring kernel^68^.

To gain anatomical specificity, significant vertex-wise statistics were described as occurring in a particular amygdala subregion. We used an anatomical reference atlas^69^ to determine the amygdala subregion in which any morphological changes occurred. Specifically, we assessed vertex-wise results in terms of their placement along both the anterior-posterior and medial-lateral axes of the amygdala, as well as their location relative to neighboring structures (e.g. hippocampus). We then matched these results to a corresponding region in the reference atlas to determine the subregion where morphological changes had occurred.

In an exploratory analysis, we also tested whether amygdala surface area differences were related to PCL-R scores among subjects with the MAOA-L genotype. These results as well as the above analyses were corrected using FDR thresholding.

### Cortical Thickness

Cortical thickness was estimated using the CIVET processing pipeline (version 1.1.12; Montreal Neurological Institute), in an application similar to previous studies^70, 71, 72^. Briefly, T1-weighted images were registered to the ICBM 152 average template with a nine parameter transformation^60^, followed by intensity inhomogeneity correction^73^ and classification into grey matter (GM), white matter (WM) and CSF^74^. Hemispheres were then modeled as GM and WM surfaces using a deformable model strategy. This approach generates four separate surfaces, each defined by 40,962 vertices^68^. Cortical thickness was determined in native space through non-linear surface-based normalization that uses a mid-surface between pial and WM surfaces. Images were then smoothed with a 20-mm surface-based diffusion kernel and non-linearly registered to a minimally biased surface-based template^75^. Native-space thicknesses were used in all analyses.

The middle and lateral orbitofrontal cortex were defined as ROIs using the cortical parcellations available in the LPBA40 atlas^76^. The intersection of atlas labels and the cortical surface allow computation of average cortical thickness measures per ROI as opposed to per vertex. The middle and lateral orbitofrontal cortex were selected a priori as regions to investigate.

### Statistics

Volumetric analyses of the amygdala and OFC were performed in the R statistical environment^77^ using a two-way (group × genotype) ANOVA to investigate the effect of the MAO-A genotype on amygdala volume in ASPD + PP offenders and healthy controls. Age, IQ, substance use, and BeAST derived total brain volumes were also included in the model. Subsequent post hoc analyses were conducted using the Tukey’s HSD method.

Vertex-wise surface area measures were analyzed with the RMINC statistical package (https://github.com/Mouse-Imaging-Centre/RMINC)^78^ using a two-way (group × genotype) ANOVA to investigate the effect of the MAO-A genotype on amygdala morphology in ASPD + PP offenders. Age, substance abuse history, and IQ were included in the model. In the event that the group × genotype interaction was significant, the interaction was analyzed by subsetting the data into MAOA-L and MAOA-H groups and running a one-way (group) vertex-wise ANOVA in each subset. All vertex-wise surface area values were divided by the total amygdala surface area to account for global structure-wise differences in total size. Vertex-wise measures were corrected for multiple comparisons using FDR^79^ as in our previous work^80^. This approach computes an FDR threshold at a given percentage for the F statistic output from the ANOVA. Therefore, among the vertices above the FDR threshold, only a certain percentage can be expected to be false positives.

### Power Analysis

Post-hoc power analyses were conducted in order to assess the statistical power of the given sample in relation to the observed means and variances. For each result, the required sample size was plotted to obtain power = 0.80 (α = 0.05) using the observed means and a range of variances.

### Data Availability

All data generated or analyzed during this study are included in this published article.
